# MMP2 and acrosin are major proteinases associated with the inner acrosomal membrane and may cooperate in sperm penetration of the zona pellucida during fertilization

**DOI:** 10.1007/s00441-012-1429-1

**Published:** 2012-05-22

**Authors:** Marvin Ferrer, Hilma Rodriguez, Lindsay Zara, Yang Yu, Wei Xu, Richard Oko

**Affiliations:** Department of Biomedical and Molecular Sciences, Queen’s University, Kingston, ON Canada K7L 3 N6

**Keywords:** Acrosome, Spermatozoa, Inner acrosomal membrane, Matrix metalloproteinase 2, Fertilization, Zona pellucida

## Abstract

Sperm-zona pellucida (ZP) penetration during fertilization is a process that most likely involves enzymatic digestion of this extracellular coat by spermatozoa. Since the inner acrosomal membrane (IAM) is the leading edge of spermatozoa during penetration and proteins required for secondary binding of sperm to the zona are present on it, the IAM is the likely location of these enzymes. The objectives of this study were to identify and characterize proteinases present on the IAM, confirm their localization and provide evidence for their role in fertilization. Gelatin zymography of detergent extracts of the IAM revealed bands of enzymatic activity identified as serine and matrix metallo-proteinases (MMPs). Specific inhibitors to MMPs revealed that MMP activity was due to MMP2. Immunoblotting determined that the serine protease activity on the zymogram was due to acrosin and also confirmed the MMP2 activity. Immunogold labeling of spermatozoa at the electron microscope level showed that acrosin and MMP2 were confined to the apical and principal segments of the acrosome in association with the IAM, confirming our IAM isolation technique. Immunohistochemical examination of acrosin and MMP2 during spermiogenesis showed that both proteins originate in the acrosomic granule during the Golgi phase and later redistribute to the acrosomal membrane. Anti-MMP2 antibodies and inhibitors incorporated into in vitro fertilization media significantly decreased fertilization rates. This is the first study to demonstrate that MMP2 and acrosin are associated with the IAM and introduces the possibility of their cooperation in enzymatic digestion of the ZP during penetration.

**Electronic supplementary material:**

The online version of this article (doi:10.1007/s00441-012-1429-1) contains supplementary material, which is available to authorized users.

## Introduction

The acrosome is a secretory vesicle that forms a cap-like structure over the anterior half of the nucleus of mammalian sperm (Gerton 2002; Tulsiani et al. 1998; Clermont and Tang 1985; Clermont et al. 1993; Tang et al. 1982; Thorne-Tjomsland et al. 1988). It is bound to the nucleus by the perinuclear theca (PT), which intervenes between the nuclear envelope (NE) and the inner acrosomal membrane (IAM) (Oko and Sutovsky 2009). The interior of the acrosome is compartmentalized morphologically and biochemically and contains a variety of proteins including several protease zymogens of which proacrosin/acrosin is the best characterized (Ohmura et al. 1999; Yu et al. 2009; Gerton 2002; Yamagata et al. 1998a).

Mammalian fertilization requires the penetration of the sperm through the zona pellucida (ZP), an extracellular coat of the egg (Yanagimachi 1994; Wassarman 1999). In order for this to occur, either passage through the cumulus oophorus, a follicular cell layer surrounding the ZP, or primary binding with the ZP induces the sperm to undergo the acrosome reaction (Wassarman et al. 2001; Yanagimachi 2011). The acrosome reaction is a form of exocytosis, where multiple fusions occur between the outer acrosomal membrane (OAM) and overlying plasma membrane allowing for the release of acrosomal contents (Gerton 2002). For many years, it was believed that the released acrosomal contents, of which acrosin was the major serine protease, would digest a path for the sperm through the ZP. However, three main experiments stand in the way of this hypothesis. First, the results of an acrosin gene knock-out showed conclusively that mouse sperm do not require acrosin for ZP penetration (Baba et al. 1994). Rather, a role for acrosin may be to accelerate the dispersal of acrosomal contents during the acrosome reaction (Yamagata et al. 1998b). Secondly, and more recently, it was observed that most fertilizing mouse spermatozoa already undergo the acrosome reaction and release their acrosomal contents in the cumulus oophorus before reaching the ZP (Jin et al. 2011). Thirdly, and most importantly, documentation of a secondary and more adhesive binding step (involving the exposed IAM and the ZP surface), occurring after the acrosome reaction and after the dispersal of acrosomal contents, precludes the possibility that the released contents predigest a path in the ZP (Mortillo and Wassarman 1991; Bleil et al. 1988). Although there are those who argue in favor of motility being sufficient to enable a sperm to push its way through the ZP (Bedford 1998), the calculated force generated by the sperm does not appear strong enough to penetrate the ZP (Green 2002) and trypsin inhibitors, which block sperm-zona penetration, do not block motility (Yamagata et al. 1998a). Furthermore, studies on the sperm proteasome now offer a plausible candidate enzyme functioning as a ZP-lysin during fertilization (Sutovsky 2011).

A prevailing concept is that, as a consequence of acrosomal exocytosis and the release of the acrosomal contents, the IAM becomes exposed allowing it to directly interact with the ZP2 protein on the ZP, a compulsory step for subsequent IAM-directed sperm penetration through the ZP (Wassarman 1988; Huang and Yanagimachi 1985; Yanagimachi 1994). The documentation of this secondary binding step by Wassarman and colleagues (Mortillo and Wassarman 1991; Bleil et al. 1988) stimulated a search for the protein receptor on the IAM that interacts with the zona. By devising a cell fractionation method that allowed us to study the protein composition of the IAM directly, we were able to identify and characterize such a receptor, termed IAM38 and reinforce the above concept (Yu et al. 2006). Since the sperm begins zona penetration only after this compulsory 'secondary' binding step, we hypothesized that the exposed IAM surface is engaged in lytic activity. Taking advantage of our sperm head fractionation procedure, by which detergent extraction can selectively solubilize IAM associated proteins, the major objective of our study was to identify and characterize proteinases on the IAM that could be candidates for enzymatic digestion of the ZP during sperm penetration. Unexpectedly, we revealed the presence of proacrosin/acrosin and matrix metalloproteinase 2 (MMP2) as IAM-associated enzymes. MMP2, also known as gelatinase A, is an MMP that is widely found in somatic tissue and is involved in the breakdown of extracellular matrix material (Werb 1997).

## Materials and methods

### Animals and ethics

The use of animals for the studies reported here was approved by the Queen’s University Institutional Animal Care and Use Committee. Ethical approval for research on the human samples in this study was obtained from the Queen's University Health Sciences Research Ethics Board.

### Sperm and sample collection and fractionation

Testicles and epididymides from six bulls on each of three different occasions were obtained at the abattoir in Joyceville (Ontario, Canada) immediately after bull slaughter. Cauda epididymides were submerged in 25 mM Tris-buffered saline (TBS), pH 7.5, containing phenylmethylsulfonylfluoride (PMSF) and cut by a razor several times to allow spermatozoa to diffuse into suspension. Spermatozoa were then separated from epididymal tissue by filtration through 80-μm nytex netting, followed by slow speed centrifugation at 1,000*g* and resuspension in TBS several times for washing. A portion of spermatozoa, resuspended in TBS containing a protease inhibitor cocktail (Complete, Mini, EDTA-free; Roche, Canada), was sonicated on ice for 3 × 15-s bursts with 1-min intervals between pulses utilizing a small probe Vibra-Cell sonicator (Sonics and Materials, Danbury, CT, USA) set at an amplitude of 40 kHz. Sonicated spermatozoa were then centrifuged at 4 °C for 10 min at 14,000*g* and the supernatant was collected. The pellet, containing separated heads and tails, was washed two times by resuspension/centrifugation in TBS, resuspended in 80 % sucrose in TBS in a 28-mL screwcap tube and centrifuged in a 55Ti angle rotor (55Ti, Beckmann, Mississauga, ON, USA) at 200,000*g* for 65 min at 4 °C. Sperm heads being denser than 80 % sucrose migrated to the centrifugal side of the tube, while tails and debris migrated to the opposite side. The centrifuge tube was turned over and emptied of sucrose before the isolated heads were collected off the side of the tube in a minimal amount of TBS followed by slow-speed centrifugation and resuspension. If the purity of the isolated head sample was <99 % (which was rare), then the heads were processed through the 80 % sucrose gradient a second time. The sonicated and isolated sperm heads (SSpH) obtained by the procedure were devoid of plasma membrane and acrosome except for the IAM that remained firmly attached to the intact PT. The same procedure as above was used successfully for the collection and isolation of murid sperm heads. Male CD1 mice 1–3 months of age were purchased from Charles River, St Constant, QC, Canada, housed under a 12-h light/dark cycle and allowed free access to food and water. Human semen was obtained from ejaculate from ten young adult male donors.

First trimester human trophoblast HTR8/SV neo cell line condition media known to contain MMP-2 and stimulated with tumor necrosis factor to induce MMP-9 expression served as a positive control for these MMPs.

### Detergent extractions

Detergents used to solubilize and extract IAM associated proteins from SSpH are as follows: non-ionic detergent Nonidet P-40 (NP-40), radioimmunoprecipitation assay buffer (RIPA) [0.1 % sodium dodecyl sulfate (SDS) and 1 % NP-40] and 1 % SDS. Bull spermatozoa were incubated with detergent solutions with continuous agitation for 2 h at 21 °C or overnight at 4 °C. Following incubation, the supernatant was separated from the pellet by centrifugation at 14,000*g* for 10 min at 4 °C and the resultant fractions were mixed with either a reducing or non-reducing sample buffer (200 mM Tris pH 6.8, 4 % SDS, 0.1 % bromophenol blue, 40 % glycerol, with or without 5 % β-mercaptoethanol) for analysis by immunoblotting or zymography, respectively.

### Zymography

Bull samples were loaded onto 10 % SDS-polyacrylamide gels containing gelatin [5.24 mg gelatin in 2.3 mL ddH_2_O, 1.25 mL 40 % acrylamide, 1.25 mL 1.5 M Tris pH 8.8, 50μL 10 % SDS, 50μL 10 % ammonium persulfate (APS), 3μL N, N, N’, N’ – tetramethylethylenediamine (TEMED)]. After electrophoresis, enzymes were renatured by rinsing twice for 30 min and once for 1 h in 2.5 % Triton X-100, 5 mM CaCl_2_, 50 mM Tris pH 7.5 in ddH_2_O at room temperature followed by overnight incubation at 37 °C in the same solution but without Triton. Trypsin and MMP gelatinase activity was studied by incubating gels with or without the MMP inhibitor GM6001 (0.2 ng/ml) (Millipore catalogue number CC1010; Billerica, MA, USA), also known as Ilomastat, or 5 μg/mL soybean trypsin inhibitor. A cyclic disulfide bonded peptide (CTTHWGFTLC) (40 μM; Calbiochem, San Diego, CA, USA) that specifically inhibits MMPs 2 and 9 but preferentially inhibits MMP2, was also used to perform preferential inhibition of MMP2 gelatinase activity (Koivunen et al. 1999). The gel was then stained with Coomassie stain and destained in 30 % methanol, 10 % glacial acetic acid and 60 % ddH_2_O for 2 h. Clear bands in the zymogram indicated enzymatic digestion of gelatin. Results shown are typical of at least three different experiments.

### Antibodies

Two different anti-MMP2 antibodies were purchased from Millipore: a monoclonal antibody (MAB3308; referred to as anti-mMMP2 in this article) that was raised against a synthetic oligopeptide corresponding to amino acid residue 468-483 of human MMP2 and a polyclonal antibody (AB19167; referred to as anti-pMMP2) raised against a synthetic peptide from the second half of human MMP-2. Both antibodies are reactive with human, rat, mouse and bovine MMP2. Both MMP2 antibodies worked equally as well on western blots but anti-pMMP2 was superior for immunocytochemistry. In-house polyclonal antibody was also raised in rabbits against an oligopeptide (aa 422-603: APIYTYTKNF RLSHDDIKGI QELYGPSPDA DTDTGTGPTP TLGPVTPEIC KQDIVFDGIA QIREIFFFKD RFIWRTVTPR DKPTGPLLVA TFWPELPEKI DAVYEAPQEE KAVFFAGNEY WVYSASTLER GYPKPLTSLG LPPDVQQVDA AFNWSKNKKT YIFAGDKFWR YNEVKKKMDP G) similar in mouse, human and bull MMP2 (referred to as anti-tMMP2, 0.88 mg/ml, preimmune IgG, 0.14 mg/ml) for use in immunoblotting, immunocytochemistry and in vitro fertilization studies. In-house polyclonal antibodies were raised in rabbits against a synthetic oligopeptide (EVEWGSNKPVKPPLQERYVEK) common to both bull and boar acrosin (referred to as anti-bull acrosin) and to the C-terminal half of recombinant mouse acrosin (referred to as anti-mouse acrosin). The anti-acrosin antibodies were affinity purified on bull and mouse recombinant acrosin and concentrated before use (1.5 mg/ml). We made our own acrosin antibodies because we found commercial sources to be less specific. For example, one popular commercial source cross-reacted with trypsin on western blots and, because there are many trypsin-like enzymes in the acrosome, it was discarded.

### Immunoblotting

Bull, human and mouse sperm and sperm head fractions before and after extractions were dissolved in sample buffer containing 2 % SDS with and without 5 % mercaptoethanol and run on 10 % SDS-PAGE according to Laemmli (1970). Proteins separated by SDS-PAGE were transferred onto polyvinylidene fluoride (PVDF) membrane (Millipore, Mississauga, ON, Canada) according to the western transfer technique proposed by Towbin et al. (1979). PVDF membranes were blocked with 10 % skim milk in phosphate-buffered saline (PBS) with 0.05 % Tween-20 (PBS-T) for 30 min at room temperature and then incubated with primary antibodies (1.5 μg/mL anti-acrosin and 1 μg/mL anti-pMMP2) in 2 % skim milk in PBS-T either at 21 °C for 2 h or overnight at 4 °C. The blots were then washed 6 × 5 min under continuous agitation and incubated with goat anti-rabbit (1:50,000) or anti-mouse (1:5,000) IgG H + L antibodies from Vector Laboratories (Burlingame, CA, USA) for 3 h at 21 °C. They were again washed 6 × 5 min in PBS-T, incubated with Pierce SuperSignal peroxide and luminol/enhancer solution from Thermo Fisher Scientific (Rockford, IL, USA) for 6 min and exposed to X-ray film for developing. Results shown are typical of at least three different samples and experiments.

### Immunohistochemistry

Bull and mouse testicular sections from testes that had been perfusion-fixed in Bouin’s fixative and embedded in paraffin were deparaffinized in xylene and hydrated through a graded series of ethanol solutions. During hydration, the sections were treated to abolish endogenous peroxidase activity, to neutralize residual picric acid and to block free aldehyde groups (Oko et al. 1996). Once hydrated, the sections were subjected to antigen retrieval by microwaving in a 0.01 M sodium citrate solution, pH 6 (Tovich et al. 2004). Immunolabeling was conducted using an avidin-biotin complex (ABC) kit (Vector Laboratories). Nonspecific sites were sequentially blocked with avidin and biotin blocking serum followed by 10 % normal goat serum (NGS) in TBS for 15 min. Primary antibody incubation followed for 2 h at 21 °C or overnight at 4 °C with anti-pMMP2 (1:3), anti-tMMP2 (1:50) or anti-bull acrosin (1:5). The anti-acrosin antibody preincubated with the peptide (2 mg/mL) it was raised against was used as a control (1:2). Normal rabbit serum or preimmune serum served as a negative control for the anti-MMP2 antibodies, respectively. After washing, 4 × 5 min in TBS containing 0.1 % Tween-20, sections were incubated with biotinylated goat anti-rabbit IgG secondary antibody followed by incubation with ABC, according to instructions from Vector Laboratories. Sections were then blocked in 10 % NGS in TBS and incubated with goat anti-rabbit IgG conjugated to peroxidase in TBS for 1 h. After washing in TBS containing 0.1 % Tween-20, peroxidase reactivity of the sections was elicited by incubating the sections with 0.03 % hydrogen peroxide and 0.05 % diaminobenzidene tetrahydrochloride in TBS containing 0.1 M imidazole, pH 7.6, for 10 min The sections were then washed with distilled H2O, counterstained with 0.1 % filtered methylene blue and immersed in tap water for 5 min. Following stain differentiation, the sections were dehydrated, cleared in xylene and mounted with coverslips using Permount. Results shown are typical of three different animals and experiments.

### Immunogold electron microscopy

Bull sperm and tissue were fixed in 4 % paraformaldehyde and 0.8 % glutaraldehyde, embedded in LR white (Canemco, St Laurens, QC, Canada), cut into ultrathin sections and mounted on formavar coated nickel grids. All solvents and solutions were double-filtered before use with P8 Fisherbrand filter paper, except for antibodies. During immunolabelling, grids were floated tissue-side down on drops of various solutions. Sections were blocked with 5 % NGS in TBS and then incubated with anti-tMMP2 (1:50) or affinity purified anti-bull acrosin (1:1) antibodies overnight at 4 ºC. The anti-acrosin antibody preincubated with the peptide (2 mg/ml) it was raised against was used as a control whereas the preimmune serum was used as a control for anti-tMMP2. They were then washed in TWBS (TBS with 0.1 % Tween-20, pH 8) extensively, blocked with 5 % NGS for 15 min, incubated in 10 nm gold particle-conjugated goat anti-rabbit IgG (Sigma, Mississauga, ON, Canada) for 2 h at 21 °C, washed with TWBS and deionized water and allowed to dry. The sections were then counterstained with uranyl acetate and lead citrate, washed with deionized water and then dried. Photographs were taken using a Hitachi 7000 transmission electron microscope. Results shown are typical of three different animals and experiments.

### In vitro fertilization of mouse oocytes

Spermatozoa from young adult male CD-1 males were squeezed out of freshly isolated mouse epididymides in Embryomax M-2 medium (Millipore catalogue number MR-015-D) using sterile forceps and allowed to ‘swim out’ for 10 min at 37 °C. The spermatozoa obtained were then overlaid with 2× volume containing Embryomax human tubal fluid (HTF) medium (Millipore catalogue number MR-070-D), with or without antibody added, for 30 min at 37 °C, 5%CO_2_. The upper 1/3 of the suspension was taken and adjusted to 5 × 10^5^ sperm/ml for fertilization. Ten-week-old CD-1 females were superovulated by intraperitoneal injection of 10 IU pregnant mare serum gonadotropin (Sigma catalogue number G4877) followed 48 h later with 10 IU human chorionic gonadotropin (Sigma catalogue number CG10). Oocytes were collected from oviducts into M-2 20 h after hCG injection and cumulus oophorus cells removed by treatment with 0.1 % hyaluronidase in M-2. The cumulus-free oocytes were washed in M2. Oocytes were pre-incubated in HTF with or without anti-pMMP2 (1:50), anti-tMMP2 (1:25), pre-immune anti-tMMP2 IgG purified antibody (1:10) or 40nM tissue inhibitor of MMP2 (TIMP2; R&D Systems 971-TM, Minneapolis, MN, USA). Pre-incubation occured for 30 min at 37 °C, 5%CO_2_ and oocytes were fertilized with spermatozoa in the same droplet at 37 °C, 5%CO_2_. Fertilization was assessed 24 h later by counting the number of zygotes as compared to oocytes. All droplets were overlaid with sterile mineral oil.

### Statistics

A chi square test was used to determine statistical significance between each category and the control group. Statistical significance is noted when *p* < 0.05.

## Results

### Gelatin zymograms reveal proteinase activity is associated with the inner acrosomal membrane

**Fig. 1:**
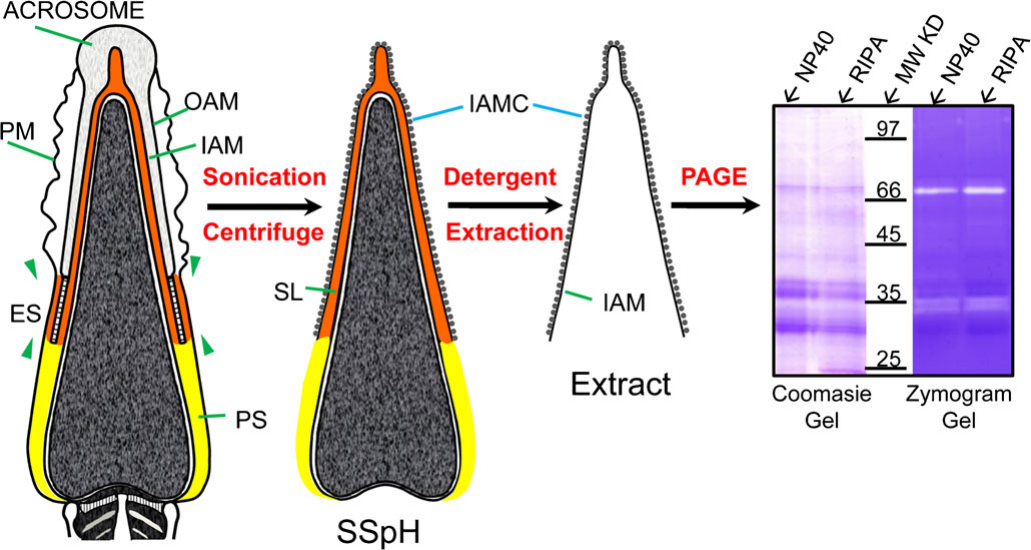
Design for detergent extraction of the inner acrosomal membrane (*IAM*, designated by a *line*) and its extracellular protein coat (*IAMC*, designated by *beads*) from sonicated and isolated sperm heads (*SSpH*). The detergent extract is separated from the SSpH by centrifugation and analyzed for protein and enzyme content by conventional and gelatin zymogram polyacrylamide gel electrophoresis (*PAGE*). Distinctive enzymatic activity is found at both the 72- and -35 kDa levels. Gel lanes were loaded with non-ionic (*NP-40*) and ionic (*RIPA*) detergent extracts, giving essentially the same results. *PM* plasma membrane; *OAM* outer acrosomal membrane; *ES* equatorial segement; *SL* subacrosomal layer (*red*); *PS* postacrosomal sheath (*yellow*). Isolation of the IAM and IAMC was based on protocol previously published by Yu et al. (2006)

**Fig. 2:**
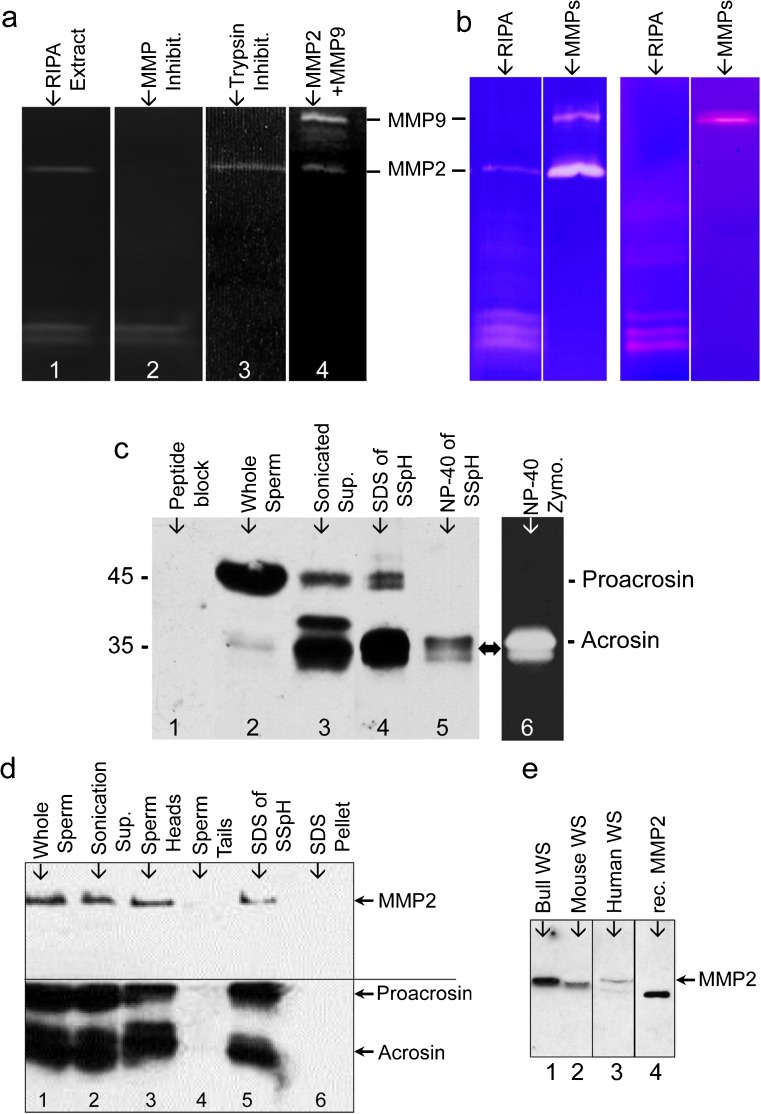
**a** Gelatin zymograms of detergent extracts (*RIPA* ) of SSpH reveal MMP and serine proteinases as components of the IAM. Blockage of the 72-kDa enzymatic activity with GM6001 (Ilomastat) (*lane 2*) indicates a metalloprotease and blockage of the 35-kDa enzymatic activities by trypsin inhibitor (*lane 3*) indicates serine proteases. *Lane 4*, a positive control, is loaded with a trophoblast cell medium containing both MMP2 (72 kDa) and MMP9 (92 kDa). **b** Gelatin zymogram without (control) and with (block) a cyclic disulfide bonded peptide (CTTHWGFTLC) on RIPA extracts of SSpH (*lane 1*) and trophoblast media (*lane 2*), containing both MMP2 and MMP9 enzymatic activities. **c** Immunoblotting verification that acrosin is responsible for serine protease activity in detergent extracts of SSpH. Anti-acrosin antibody detects proacrosin in western blots of whole bull epididymal sperm (*lane 2*) and is blocked when preincubated with the peptide it was raised against (*lane 1*). Sonication of whole sperm causes proacrosin cleavage into its active forms as indicated both in the resultant sonication supernatant (*lane 3*) and in SSpH (*lane 4*). The 2 % NP-40 (non-ionic detergent) extract of SSpH (*lane 5*) is less efficient in stripping SSpH of proacrosin/acrosin than 2 % SDS (*lane 4*). *Lane 6* is a gelatin zymogram of the NP-40 extract loaded in *lane 5*, confirming that the enzymatic activities found at the 35-kDa level are due to acrosin. **d** Immunoblotting verification that both MMP2 and Proacrosin/Acrosin are constituents of the sonicated bull sperm head. Freeze–thawed sperm (*lane 1*) were sonicated and separated by centrifugation into three fractions: supernatant (*lane 2*), SSpH (*lane 3*), and tails (*lane 4*). The sperm heads were then extracted with SDS (*lane 5*) and compared with pellet (*lane 6*). The upper part of the western blot was probed with anti-pMMP2, while the bottom part below the demarcating *line* was probed with polyclonal anti-bull acrosin antibody. **e** Immunoblots probed with anti-tMMP2 showing that MMP2 is present in bull, mouse and human spermatozoa (WS). Human recombinant MMP2, minus the pre-domain, is used as a positive control (rec. MMP2)

In order to study the protein composition of the IAM; we previously devised a cell fractionation procedure (Yu et al. 2006) by which the IAM and its extracellular protein coat (IAMC) were exposed on the surface of isolated sperm heads (Fig. 1). Detergent extraction of this sonicated and sucrose gradient isolated sperm head (SSpH) fraction solubilized the proteins of the IAM and its IAMC but left the detergent resistant nucleus and PT intact (Fig. 1). The detergent soluble proteins were collected in the supernatant after centrifugation and analyzed by PAGE and gelatin zymography for protein and enzymatic content (Fig. 1). Gelatin zymograms of NP-40 and RIPA extracts of bull SSpH revealed two intense zones of enzymatic activity (clear bands) at approximately 35- and 72-kDa levels (Fig. 1). Co-incubation of the extract loaded gelatin gel with the MMP inhibitor Ilomastat removed the 72-kDa clear band, confirming that this enzymatic activity was due to a matrix metalloproteinase (MMP) while co-incubation of the gelatin gel with a trypsin inhibitor removed the clear bands at the 35-kDa level, confirming that these enzymatic activities were due to serine proteases (Fig. 2a). A gelatin zymogram loaded with a trophoblast cell extract, containing both MMP2 and MMP9, was also analyzed as a positive control for MMP activity and suggested that the 72-kDa enzymatic activity in the RIPA-SSpH extract may be due to MMP2 (Fig. 2a, lane 4).

### Specific inhibition of MMP2 activity associated with the IAM

Co-incubation of the zymogram loaded with RIPA extracts of bull SSpH with a cyclic disulfide bonded peptide (CTTHWGFTLC), a specific inhibitor of MMP2 and MMP9 that preferentially inhibits the activity of MMP-2 relative to the activity of MMP-9 (Koivunen et al. 1999), inhibited the 72 kDa MMP-enzymatic activity confirming the presence of MMP2 at this level (Fig. 2b). Trophoblast cell media, containing both MMP2 and MMP9, served as a negative control demonstrating the efficacy of the inhibitor. CTTHWGFTLC preferentially inhibited MMP2- over MMP9-activity (Fig. 2b) indicating that the inhibition was specific.

### Acrosin is responsible for the serine protease activity associated with the IAM

In order to show that the serine protease activity associated with the detergent extract of the SSpH is due to acrosin, we raised an antibody against a specific peptide region common to both bull and boar acrosin. This anti-bull acrosin antibody appeared specific as it predominantly labeled a 43-kDa band in freshly isolated or ejaculated bull sperm (Fig. 2c, lane 2), corresponding to the expected size of proacrosin. Furthermore, this immuno-reactivity along with a more minor immunoreactivity at the 35-kDa level was inhibited when the anti-bull acrosin antibody was preincubated with the peptide it was raised against (Fig. 2c, lane 1). Sonication of fresh bull sperm before immuno-blotting analysis resulted in a shift of the immunoreactivity to the 35-kDa level in the supernatant (lane 3) as well as in the SSpH (lane 4), which only retained the IAM part of the acrosome. This shift was expected as sonication breaks up the acrosome and initiates cleavage of proacrosin to acrosin, just as the acrosome reaction does (Brown and Harrison 1978). Importantly, there was almost an equal amount of acrosin immunoreactivity retained in the SSpH as in the supernatant after sonication (compare lanes 3 and 4). This indicates that a sizeable portion of proacrosin/acrosin is retained on the IAMC. Extraction of the SSpH with non-ionic detergent, NP-40, was not as efficient in getting acrosin off the IAM as with ionic detergent, SDS, (compare lanes 4 and 5). This suggests that there may be an inter-linkage through the IAM between the PT and the IAMC. Nevertheless, comparison of the immunoblot (lane 5) and gelatin zymogram (lane 6), both equally loaded with NP-40 extract, showed that the acrosin-immunoreactive bands corresponded in molecular mass and intensity to the enzymatic digested bands, indicating that the digestive activity was due to acrosin.

### Comparison of MMP2 and proacrosin/acrosin immunoreactivity in sonicated sperm fractions

The presence of MMP2 as a 72-kDa band was confirmed on immunoblots of the sonicated supernatant of whole bull sperm, bull SSpH and SDS extracts of bull SSpH by utilizing a commercial monoclonal anti-mMMP2 antibody (not shown), as well as a commercial polyclonal antibody (anti-pMMP2) raised against a peptide sequence of MMP2 (Fig. 2d). Both antibodies were found to be monospecific but the latter was chosen for immunocytochemistry as it was found to be more antigenic. Importantly, there was almost an equal amount of MMP2 immunoreactivity retained in the SSpH as in the supernatant after sonication (compare lanes 2 and 3) indicating that a sizeable portion of MMP2 is retained on the IAMC. SDS was able to completely extract MMP2 from the SSpH (compare lanes 5 and 6).

Since MMP2 (72 kDa) and proacrosin (43 kDa) and acrosin (35 kDa) are very different sizes, the same blot used to immunoprobe for MMP2 (above) was also used to probe for acrosin/proacrosin (Fig. 2d). The similar immunoblotting profile between these proteins to different cellular fractionation conditions like sonication and detergent extraction reinforces the idea that MMP2 and proacrosin/acrosin have similar cellular locations and membrane binding properties within the sperm acrosome. Interestingly, the conversion of proacrosin to acrosin did not appear as efficient after sonication of freeze-thaw sperm (Fig. 2d) as with fresh sperm (Fig. 2c).

MMP2 was also immunodetected in human and mouse sperm (Fig. 2e). Zymograms of sperm fractions in these species confirmed MMP2 activity (not shown).

### Immunogold localization of acrosin and MMP2 confirms IAM association

**Fig. 3:**
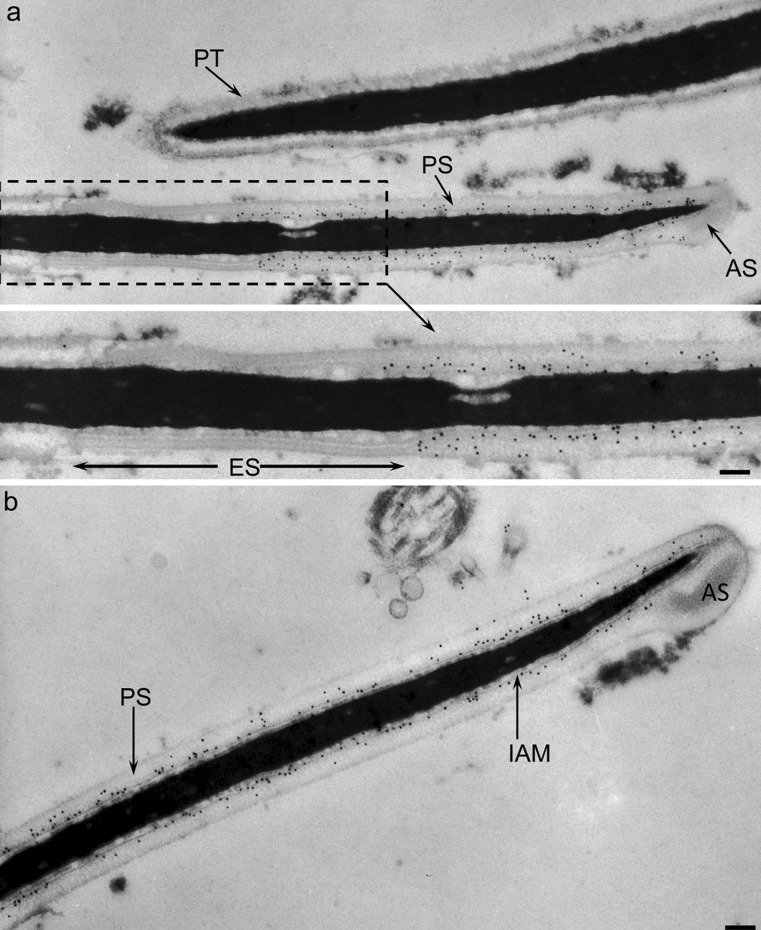
Immunogold localization of acrosin in sagittal sections of bull ejaculated sperm, utilizing anti-bull acrosin antibody. **a** Labeling is confined to the principal (*PS*) and apical (*AS*) segments of the acrosome. Little or no labeling is evident in the equatorial segment (*ES*) of the acrosome nor in the perinuclear theca (PT). **b** Labeling is closely associated to the IAM. *Bars* 0.2 μm. See Suppl. Fig. 1a for peptide blocking control

**Fig. 4:**
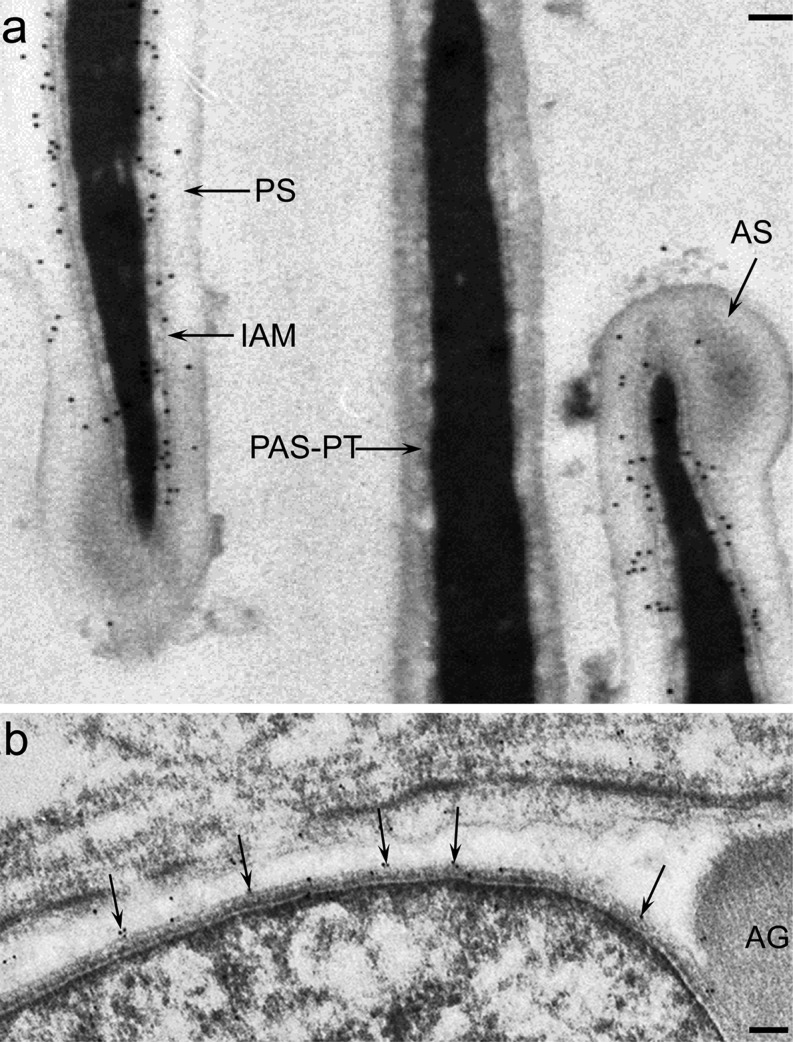
Immunogold localization of MMP2 in bull spermatozoa and spermatid at end of cap phase. **a** In ejaculated sperm immunogold labeling, utilizing anti-tMMP2 antibody, is found through the apical (*AS*) and principal segments (*PS*) of the acrosome, a large portion associated with the IAM. See Suppl. Fig. 1b for preimmune control. Bar 0.2 μm. **b** In step 7-8 spermatid labeling with anti-pMMP antibody is seen along the inner acrosomal membrane (*arrows*) of the acrosome. *AG* acrosomic granule. *Bar* 0.2 μm

In order to confirm our biochemical fractionation data that acrosin and MMP2 were associated with the IAM, we performed immunogold labeling of bull spermatozoa at the electron microscope level utilizing polyclonal antibodies we raised against a peptide sequence in bull acrosin (anti-bull acrosin) and against a portion of a sequence in mouse MMP2 (anti-tMMP2). Acrosin was mostly confined to the principal and apical segments in association with the IAM (Fig. 3). The immunogold labeling was eliminated when the antibody was pre-incubated with the peptide it was raised against prior to its use (see suppl. Fig. 1). This IAM association, within the principal and apical segments of the acrosome, was also found for MMP2 (Fig. 4).

### The acrosomal membrane association of acrosin and MMP2 begins early during spermiogenesis

**Fig. 5:**
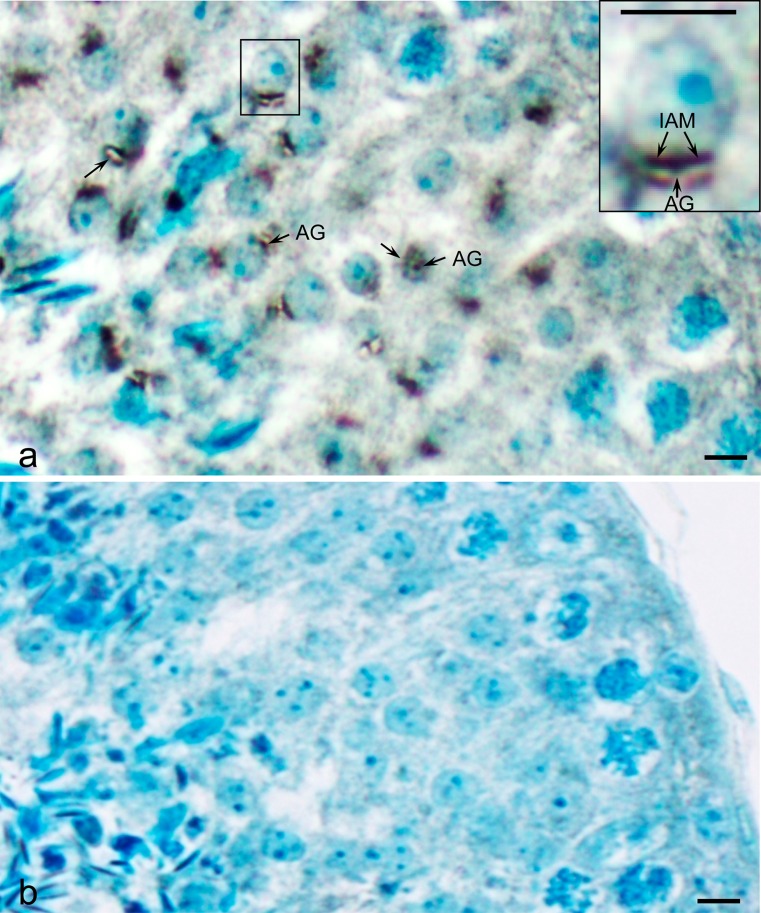
**a** Immunoperoxidase staining of proacrosin/acrosin in a testicular section of round spermatids in step 4 of bovine spermiogenesis. Immunostaining arises in the acrosomic granule (*AG*) from where it gradually shifts during the cap phase of spermiogenesis to the acrosomal membrane (*arrow*), especially to the IAM. **b** Immunoperoxidase control (step 4) in which the anti-bull acrosin antibody was pre-incubated with the peptide it was raised against prior to its use. *Bars* 5 μm

**Fig. 6:**
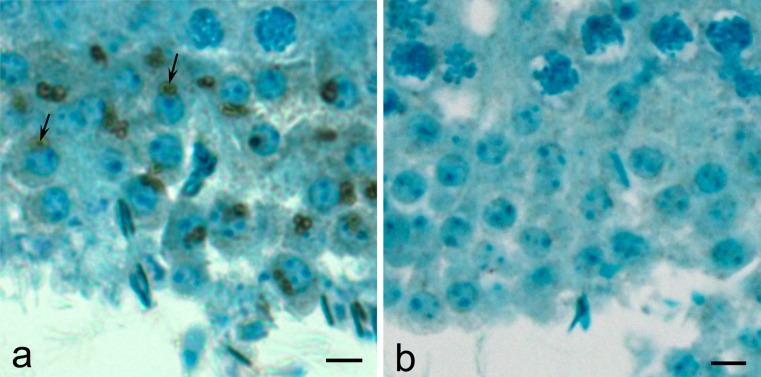
Immunoperoxidase staining of MMP2 (with anti-tMMP2 antibody) in a testicular section of round spermatids in step 2–3 of bovine spermiogenesis. **a** As with acrosin, MMP2 immunostaining is found in the acrosomic granule (*arrows*) of proacrosomic and acrosomic vesicles before shifting to the acrosomal membrane during the cap phase of spermiogenesis. **b** Preimmune control. *Bars* 5 μm

**Fig. 7:**
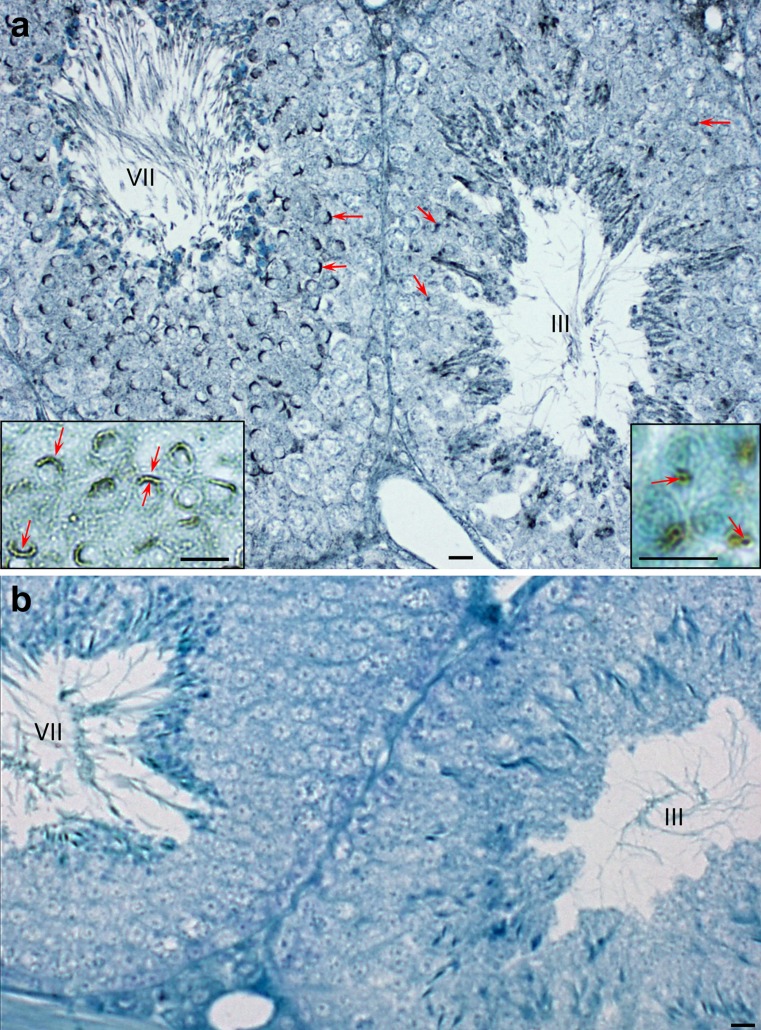
Immunmoperoxidase staining of MMP2 in stages III and VII of the cycle of mouse seminiferous epithelium. **a** MMP2 immunostaining, utilizing anti-pMMP2 antibody, is associated with the acrosomic vesicles (*arrows*) of step 3 spermatids in stage III and the acrosomic cap of step 7 spermatids (*arrows*) in stage VII. The association of MMP2 with the acrosomic granule (*arrows*) of step 3 spermatids and the acrosomal membrane (*arrows*) of step 7 spermatids is seen to have a better advantage at higher magnification in the insets. *Bars* 10 μm. **b** Normal rabbit serum control

In order to get a rudimentary understanding of the origin and assembly of acrosin and MMP2 during spermatogenesis, immunoperoxidase staining utilizing anti-bull acrosin and anti-anti-tMMP2 was first performed on bull testicular tissue fixed in Bouin’s and embedded in paraffin. Early in spermiogenesis at the beginning of the cap stage, anti-acrosin staining was already seen associated with the acrosomal membranes, appearing more intensely associated with the IAM than the OAM (Fig. 5)., There was little immunostaining evident between these membranes, except within the acrosomal granule (Fig. 5). Anti-MMP2 staining followed a similar pattern. During the Golgi phase of spermiogenesis, it was more intense over the acrosomic granule of the proacrosomic and acrosomic vesicles than over the acrosomal membrane (Fig. 6). Later, in the cap phase, staining became more intense over the acrosomal membrane, especially over the IAM as shown in the mouse (Fig. 7). Mouse testis immunostained with anti-pMMP2 had a similar MMP2-staining profile to bull testis. During the Golgi phase, MMP2 immunostaining was associated with the proacrosomic and acrosomic vesicles. As shown at higher magnification and resolution (Fig. 7, right inset), it was most intense over the acrosomal granule. In the cap phase, the MMP2 immunostaining shifted from the acrosomic granule to the acrosomal membrane (Fig. 7, left inset). Immunogold labeling confirmed that MMP2 was associated with the IAM by the end of the cap phase (see Fig. 4b).

### Inhibition of MMP2 during IVF significantly decreases fertilization

**Fig. 8:**
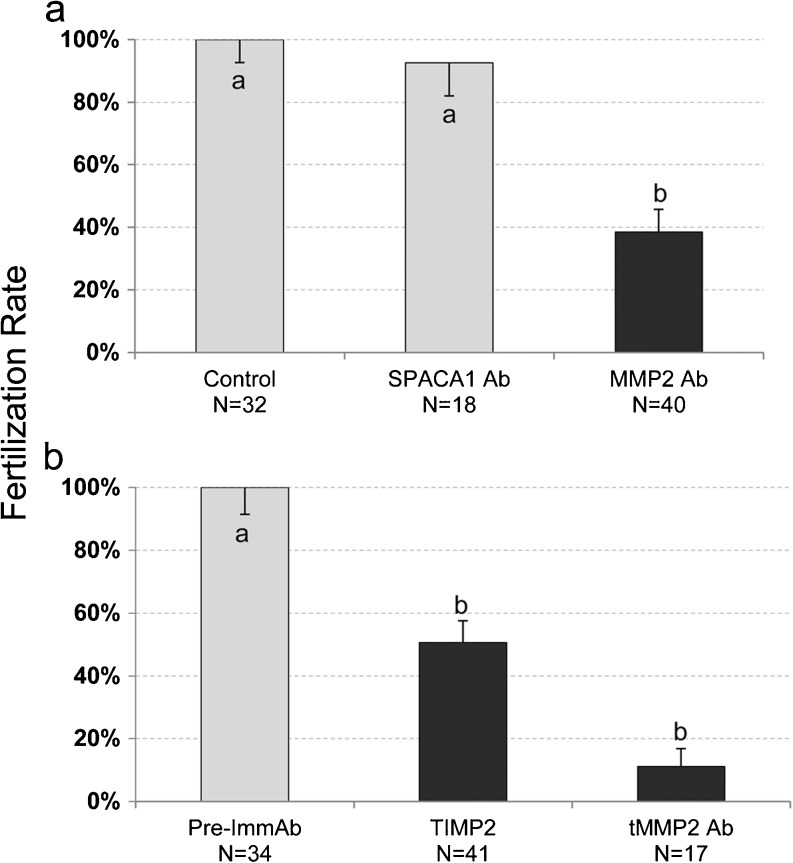
Effect of anti-MMP2 antibodies and TIMP2 in mouse IVF. IVF control values were adjusted to 100 % for comparative purposes and experimental values obtained were adjusted accordingly. The *block graphs* depict the mean percentage of oocytes that were fertilized after incubation with sperm in IVF medium containing anti-pMMP2, anti-SPACA1, anti-tMMP2, or TIMP2, as compared to controls. Superscripts *a* and *b* denote a significant difference at *P* < 0.05 and *error bars* indicate standard error. In (**a**), only the pre-incubation medium with spermatozoa contained antibodies of which only part was transferred to the final IVF medium. In (**b**) both spermatozoa and oocytes were preincubated with antibodies and inhibitors that were combined in the final IVF medium. Thus, it is difficult to distinguish if the difference in the effectiveness of inhibition in (**a**) and (**b**) is due to anti-MMP2 antibody effectiveness or concentration of antibody in IVF media

Since the IAM is the leading edge of the sperm during zona pellucida penetration, we hypothesized that MMP2 may be involved in zona-digestion as this enzyme resides on the IAM. To test this hypothesis, we attempted to block mouse fertilization in vitro by applying MMP2 inhibitors and anti-MMP2 antibodies (anti-pMMP2 and anti-tMMP2) to the IVF medium. Spermatozoa exhibited a significant reduction in the ability to fertilize the oocyte when either of the antibodies was added to the fertilization media. Control IVF medium contained no antibody added or the addition of only preimmune anti-serum for anti-tMMP2 antibody (Fig. 8). To further verify the fertilization blocking specificity of the anti-pMMP2 antibodies, they were replaced by an antibody against an unrelated but IAM-associated, protein, SPACA1, which was found to be ineffectual (Fig. 8). Finally, a specific tissue inhibitor of matrix metalloproteinases, TIMP2, which has a preference for MMP2, was also found to significantly inhibit fertilization when added to the IVF medium.

## Discussion

The controversy whether proteinases such as acrosin are associated with the IAM has been debated for many years (Huang and Yanagimachi 1985). By utilizing a sperm fractionation methodology to obtain direct information on the protein composition of the IAM, we were able to show conclusively that the IAM harbors not only ZP receptor, IAM38 (Yu et al. 2006) but also two unrelated proteinases (this study): a serine protease, acrosin/proacrosin and a matrix-metalloproteinase, MMP2. The validity of this fractionation technique for identifying IAM-associated proteins was confirmed by EM immunogold localization of IAM38 (Yu et al. 2009) and of acrosin and MMP2 (this study) to the IAM of elongated spermatids and spermatozoa.

Our study is first to identify MMP2 as an IAM-associated protein. An earlier study identified MMP2 and MMP9 activities in human spermatozoa and by immunofluorescence, localized MMP2 mainly to the acrosomal region of the sperm head and MMP9 mainly to the mid-piece of the sperm tail (Buchman-Shaked et al. 2002). Matrix metalloproteinases (MMPs) are zinc-dependent endopeptidases that contribute to physiological tissue invasion by cleaving extracellular matrix constituents (i.e., collagens, laminin, fibronectin and proteoglycans) at the leading edge of the invading cells (Hrabec et al. 2007). For example, early in the first trimester of pregnancy, human embryo implantation of the uterine wall is dependent on MMP-2 as the main gelatinase and enzyme in trophoblast invasion of the uterine stroma (Staun-Ram et al. 2004). Later in the first trimester, both MMP-2 and -9 participate in trophoblast invasion. MMP2 is also implicated in non-physiological tissue invasion, allowing tumor cell invasion and metastasis in breast (Jezierska and Motyl 2009), lymph (Tokuraku et al. 1995), ovarian (Kenny et al. 2008) and colorectal (Murnane et al. 2009) cancers, as well as in others. Thus, the idea that MMP2, a type IV collagenase, could play a role in lytic digestion of the ZP by the sperm is not surprising considering its wide range of cleavage site motifs and substrates (Dean and Overall 2007) and that normally, like all MMPs, it is secreted at the cell surface where it is activated to aid in invasion and regulation of the extracellular matrix (Hulboy et al. 1997; Nagase and Woessner 1999). In fact, an alternative to the trypsin or acidified Tyrode’s digest methods of preparing ZP-free oocytes is by pre-incubation of oocytes in type I collagenase (Yamatoya et al. 2011).

Our immunolocalization of acrosin at the electron microscope level is distinct from previous studies (Tesarik et al. 1988; Castellani-Ceresa et al. 1983; Johnson et al. 1983; Huneau et al. 1984) in that our labeling is confined to the apical and principal segments of the bull acrosome in association with the IAM. It is not scattered through the acrosomal matrix of the apical and principal segments, associated with the outer acrosomal membrane, nor found in the equatorial segment. The differences in the localization of acrosin found between our study and others may have arisen from different states of sperm preservation or manipulation prior to fixation and/or to the use of non-specific antibodies to acrosin. Most previous evaluations were done on sperm undergoing spontaneous or chemically induced acrosome reactions, while in our analysis the spermatozoa were fixed in situ in the cauda epididymis or fixed immediately after ejaculation. Nevertheless, most of the EM studies above showed that at least a portion of acrosin/proacrosin is associated with the IAM. In our sperm fractionation studies, we found that sonication was able to disrupt approximately half of the amount of acrosin within the acrosome while the other half was retained on the IAMC. Interestingly, even though a sizeable portion of acrosin/proacrosin is sonication resistant, most of it, as probed by immunofluorescence using our anti-bull acrosin antibody, appeared to have dissipated away with the acrosomal shroud on the surface of the ZP during IVF-induced acrosome exocytosis (Sutovsky and Oko, unpublished observation), agreeing with the observations of Tesarik et al. (1988). Therefore, the question remains: is there enough acrosin retained on the IAM after the acrosomal reaction in vivo to be involved in lytic digestion of the ZP during sperm-penetration; alternatively, in accelerating the dispersal of acrosomal contents during the acrosome reaction (Yamagata et al. 1998b), is acrosin involved in the activation of other proteinases found on the IAM that are involved in lysis of the ZP.

Our finding that matrix metalloproteinase 2 and acrosin are found together on the IAM and that inhibition of MMP2 enzymatic activity significantly blocks fertilization, raises the possibility that acrosin may potentially activate MMP2. MMP2, like all MMPs, is synthesized as an inactive zymogen (pro-MMP2), which requires cleavage for activation to its active form (Nagase and Woessner 1999). MMP2 contains three domains, with the first being absent from the active form as it is the pre-domain. Normally, in its somatic environment, pro-MMP2 is cleaved by membrane-type MMP1 (MT1-MMP) (Tokuraku et al. 1995; Kazes et al. 2000), an integral membrane protein (Sato et al. 1994), in complex with tissue inhibitor of MMP2 (TIMP2) (Kazes et al. 2000; Sato and Takino 2010), as well as a variety of other MMPs (Jezierska and Motyl 2009). Interestingly, trypsin also possesses the catalytic and regulatory ability to activate pro-MMP2 (Lindstad et al. 2005). For MMP2 to be functionally relevant in the acrosome, its activators must be present and, like proacrosin, it must be activated during or after the acrosome reaction. Since acrosin has trypsin-like activity and various trypsin inhibitors block in vitro fertilization (Fraser 1982; Miyamoto and Chang 1973; Saling 1981), the possibility arises that the delay in sperm penetration of the ZP seen in acrosin-null mice during IVF (Baba et al. 1994; Adham et al. 1997) is not due to acrosin’s failure to lyse the ZP. It may rather be due to its failure to cleave IAM-associated pro-MMP2 into its active form, which would affect the efficiency of its conversion and hence ZP-lysis. However, we are unable to conclusively determine at this point if the deficiency seen in IVF upon MMP2 inhibition is due to its effects on a mechanism other than ZP-lysis, such as acrosomal dispersal. Even though acrosin may be in the most favorable position on the IAM to activate MMP2, there are several other trypsin-like enzymes, such as the TESP family members (Honda et al. 2002; Kohno et al. 1998), as well as other serine proteases present within the acrosome that could potentially compensate for a loss in acrosin activity. Because some proprotein convertases have MMP activation ability (Yana and Weiss 2000), a promising candidate could be proprotein convertase subtilisin/kexin-like 4 (PCSK4, also known as serine protease prohormone convertase 4, or PC4), whose inactivation causes severe male subfertility (Gyamera-Acheampong et al. 2006). Although localized to the acrosome region, its exact location and function have not yet been resolved. Another serine protease, plasmin/plasminogen, can activate pro-MMP2 in somatic cells in a membrane-dependent manner (Monea et al. 2002) and is present in the ZP of hamster oocytes (Jimenez-Diaz et al. 2002). Plasminogen activators were identified in the acrosome of bull and human spermatozoa (Smokovitis et al. 1992) and are released during the acrosome reaction (Taitzoglou et al. 1996), while plasminogen activator receptor, SAMP14, was localized to the acrosome and retained on the IAM after the acrosome reaction (Shetty et al. 2003), suggesting that plasmin could also be a candidate MMP activator in sperm. Indeed, the addition of plasmin to IVF medium significantly decreased zona pellucida solubilization time and improved IVF success rates at certain doses (Sa et al. 2006).

Unlike the sonication-induced conversion of pro-acrosin to acrosin, the MMP2 retained on the IAM of SSpH remained mostly in its pro form. In this state, it is well known that MMP2 can still digest gelatin in zymograms. This indicates that pro-MMP2 may require a longer incubation period with its activators than sonication provides. Alternatively, its activation may be dependent on activators present in the external milieu of the female reproductive tract, outside the confines of the acrosome. The two reasons given may not be mutually exclusive, because in double-knockout mice lacking two major acrosomal serine proteases, acrosin and PRSS21(TESP5), the female reproductive tract was able to compensate for the loss of the sperm function (Kawano et al. 2010). In other words, the acrosin- and PRSS21-deficient sperm were unable to fertilize in vitro unless uterine fluid was added to the IVF media. It would not be unreasonable to assume that the uterine fluid contains factors that contribute to MMP activation, such as plasminogen and that this ‘reproductive tract’ compensation could therefore be dependent on conversion of pro-MMP2 on the exposed inner surface of the sperm to its active form. This could be easily tested for by adding plasminogen or plasmin to the IVF medium to see if it would compensate for the IVF inefficiency of acrosin- and PRSS21-deficient sperm. If this scenario proved true, then one could assume that, under normal IVF conditions, sperm-borne trypsin-like serine proteases, such as acrosin and PRSS21, would compensate for the lack of uterine fluid and activate MMP2 .

Although not a high resolution microscopic developmental study, our immunoperoxidase evaluation of MMP2 and acrosin on testicular sections shows that these enzymes are first incorporated as part of the acrosomal granule during the Golgi phase of spermiogenesis, followed by a gradual shift from the acrosomal granule to the acrosomal membrane during the cap and elongating phases. The similarities in the developmental aspects of MMP2, acrosin and IAM38 (Yu et al. 2009; Ferrer et al. 2012) support the hypothesis that peripherally attached acrosomal membrane proteins like acrosin and MMP2, as distinct from integral membrane proteins such as SPACA1 (Ferrer et al. 2012; Hao et al. 2002), follow a similar pattern of acrosomal incorporation. Certainly, by the time of spermatid maturation and production of spermatozoa, our EM immunolocalization studies indicate that all three peripheral membrane proteins (i.e., MMP2, acrosin and IAM38) end up associated with the IAM. Interestingly, although IAM38 is associated with the IAM in the apical and principal segments of the acrosome, as are MMP2 and acrosin, it differs from the latter two enzymes in that it extends into the equatorial segment associating with both the IAM and OAM in this region (Yu et al. 2006, 2009). Since IAM38/ZPBP2 is involved in acrosomal compaction during spermiogenesis (Lin et al. 2007), it is quite possible that this positioning of IAM38 allows for the narrowing of the equatorial segment that occurs during the maturation phase of spermiogenesis (Yu et al. 2009).

On consideration of the strategic developmental positioning of MMP2 and acrosin on the IAM, it is probable that the exposure of these two enzymes, along with IAM38, on the surface of the sperm after acrosomal exocytosis may be to aid the sperm in binding and penetrating the ZP of the oocyte. Their co-localization suggests that they may cooperate in sperm-zona penetration.

## Electronic Supplementary Material

Below is the link to the electronic supplementary material.

Supplementary Fig. 1 Blocking (a) and Pre-immune (b) control sections of bull spermatozoa for anti-bull acrosin antibody (Fig. 3) and anti-tMMP2 antibody (Fig. 4a), respectfully. AS, apical segment of acrosome; PS, principal segment of acrosome; ES, equatorial segment of acrosome; PAS-PT, postacrosomal sheath of PT. Bars, 0.2 (JPEG 59 kb)

High resolution image (TIFF 8704 kb)
