# Supplementary figures and images for: MMP2 and acrosin are major proteinases associated with the inner acrosomal membrane and may cooperate in sperm penetration of the zona pellucida during fertilization
Source: Cell Tissue Res. 2012 May 22;349(3):881–95. doi: 10.1007/s00441-012-1429-1 (PMC3429778; doi:10.1007/s00441-012-1429-1)

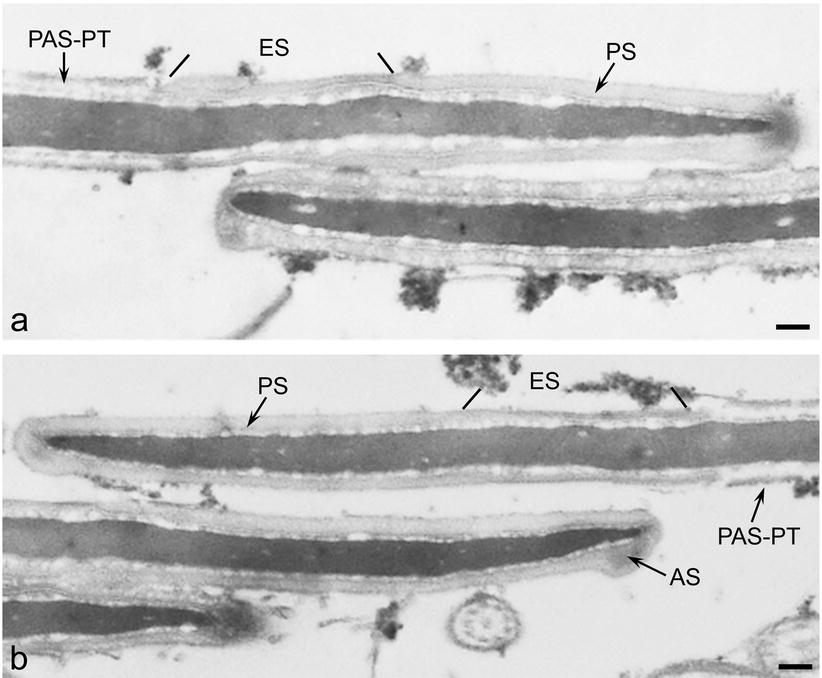

Supplement: Supplementary file 1 — Blocking (a) and Pre-immune (b) control sections of bull spermatozoa for anti-bull acrosin antibody (Fig. 3) and anti-tMMP2 antibody (Fig. 4a), respectfully. AS, apical segment of acrosome; PS, principal segment of acrosome; ES, equatorial segment of acrosome; PAS-PT, postacrosomal sheath of PT. Bars, 0.2 (JPEG 59 kb) [file 441_2012_1429_Fig9_ESM.jpg]

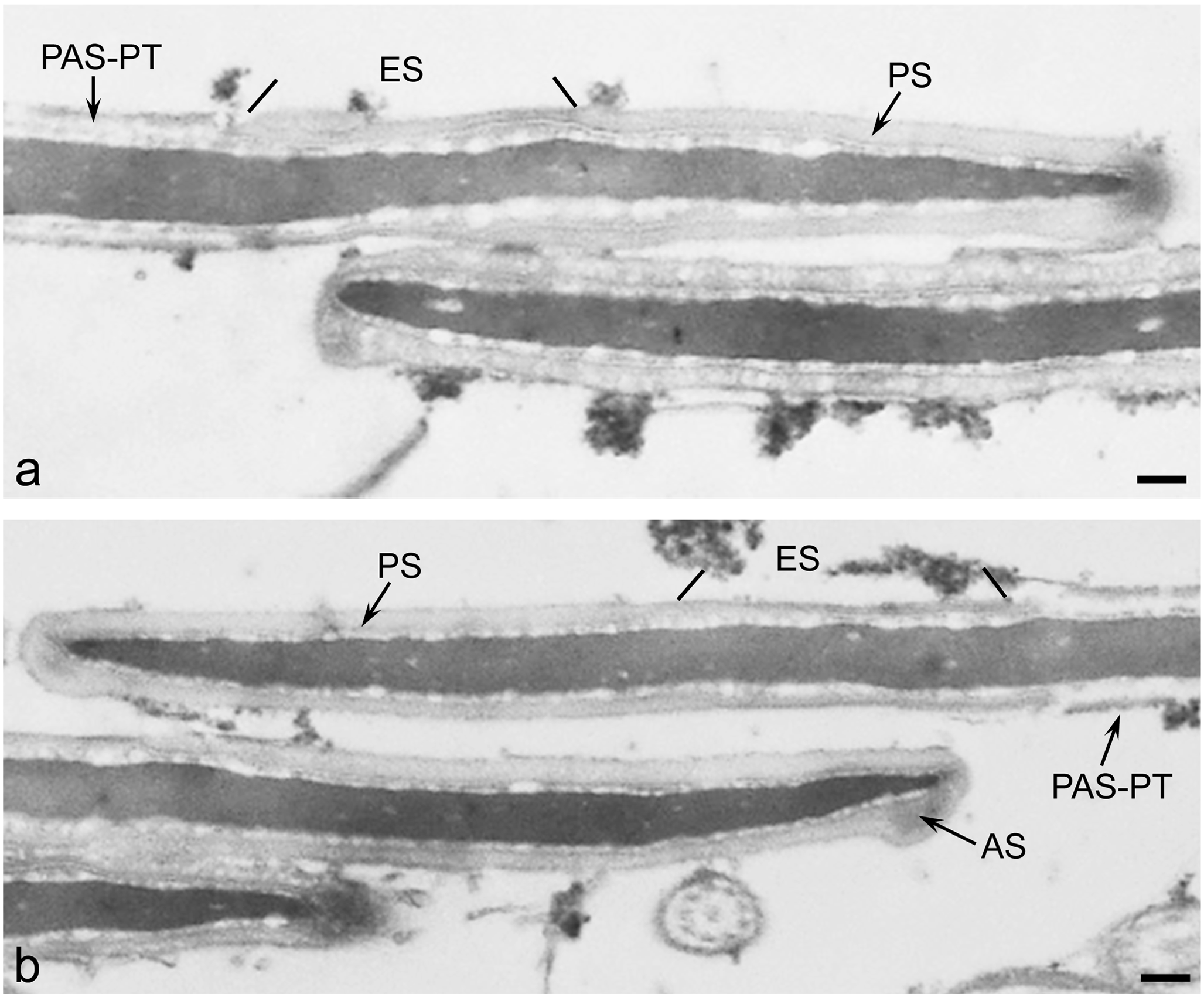

Supplement: Supplementary file 2 — High resolution image (TIFF 8704 kb) [file 441_2012_1429_MOESM1_ESM.tif]
